# Immune‐related adverse events in non‐small cell lung cancer: Occurrence, mechanisms and therapeutic strategies

**DOI:** 10.1002/ctm2.1613

**Published:** 2024-03-07

**Authors:** Xuwen Lin, Mei Xie, Jie Yao, Xidong Ma, Lin Qin, Xu‐Mei Zhang, Jialin Song, Xinyu Bao, Xin Zhang, Yinguang Zhang, Yiming Liu, Wenya Han, Yiran Liang, Ying Jing, Xinying Xue

**Affiliations:** ^1^ Department of Respiratory and Critical Care Emergency and Critical Care Medical Center Beijing Shijitan Hospital Capital Medical University Beijing China; ^2^ Department of Respiratory and Critical Care Chinese PLA General Hospital Beijing China; ^3^ Department of Endoscopic Diagnosis and Treatment Tuberculosis and Thoracic Tumor Institute Beijing Chest Hospital Capital Medical University Beijing China; ^4^ Department of Pathology Affiliated Hospital of Weifang Medical University Weifang Shandong China; ^5^ Department of Respiratory and Critical Care Shandong Second Medical University Shandong China; ^6^ Department of Thoracic Surgery Beijing Tiantan Hospital Capital Medical University Beijing China; ^7^ Department of Thoracic Surgery Chinese PLA General Hospital Beijing China; ^8^ Department of Respiratory and Critical Care Taihe Hospital Hubei University of Medicine Shiyan China; ^9^ Center for Intelligent Medicine Greater Bay Area Institute of Precision Medicine (Guangzhou) School of Life Sciences Fudan University Guangzhou Guangdong China

**Keywords:** immune checkpoint inhibitors, immune‐related adverse events, mechanism, non‐small cell lung cancer

## Abstract

The emergence of immune checkpoint inhibitors (ICIs) has heralded a transformative era in the therapeutic landscape of non‐small cell lung cancer (NSCLC). While ICIs have demonstrated clinical efficacy in a portion of patients with NSCLC, these treatments concurrently precipitate a spectrum of immune‐related adverse events (irAEs), encompassing mild to severe manifestations, collectively posing a risk of significant organ damage. Consequently, there exists an imperative to augment our comprehension of the pathophysiological underpinnings of irAEs and to formulate more efficacious preventive and ameliorative strategies. In this comprehensive review, we delineate the clinical presentation of organ‐specific irAEs in patients with NSCLC and provide an in‐depth analysis of recent advancements in understanding the mechanisms driving ICI‐induced toxicity. Furthermore, we discuss potential strategies and targets for ameliorating these irAEs. Ultimately, this review aims to furnish valuable insights to guide further research endeavours in the context of irAEs in NSCLC patients.

## Graphical abstract

- Immunotherapy not only revolutionises lung cancer treatment but also poses serious toxicity risks.
- Elucidating irAE mechanisms in NSCLC: T/B‐cell dysregulation, cytokine imbalances, autoantibody production, genetic predispositions and gut microbiome alterations.
- Highlighting precise irAE interventions in NSCLC: corticosteroids, targeted immunosuppressants, monoclonal antibodies targeting lymphocytes, cytokine inhibitors and signalling pathway modulators.

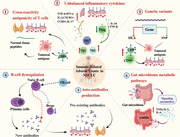

## 1 BACKGROUND

Non‐small cell lung cancer (NSCLC), accounting for 80%–85% of lung cancer cases, often reaches an advanced diagnosis stage, resulting in a bleak prognosis for approximately 70% of patients.^1^ The 5‐year survival likelihood in NSCLC patients with advanced stage barely exceeds 3%.^1^ In recent years, immune checkpoint inhibitors (ICIs) such as cytotoxic T‐lymphocyte antigen 4 (CTLA‐4), programmed death‐1 (PD‐1) and programmed death‐ligand 1 (PD‐L1) inhibitors have revolutionised the treatment landscape of NSCLC.^2^, ^3^, ^4^, ^5^, ^6^ For example, NSCLC patients treated with atezolizumab in the IMpower110 trial exhibited improved median overall survival (OS) relative to chemotherapy (20.2 vs. 13.1 months).^7^

However, immunotherapy can induce diverse adverse effects, referred to as immune‐related adverse events (irAEs), that resemble autoimmune pathologies.^8^ As demonstrated in numerous clinical trials, irAEs induced by immunotherapy primarily manifest as impairments in the cardiac, respiratory, gastrointestinal, skin and neurological systems.^8^, ^9^, ^10^ In several clinical trials, 45%–60% of NSCLC patients treated with atezolizumab experienced irAEs.^11^, ^12^, ^13^ A systematic analysis encompassing 16 clinical studies reported an overall irAE incidence of 22%, with severe irAEs (grade 3–4) affecting 4% of NSCLC patients under anti‐PD‐1/PD‐L1 treatment.^9^ While most cases can be effectively managed, a subset may present with moderate to severe toxicities, thereby severely compromising organ functionality.^14^

Given the frequency and severity of irAEs, understanding their underlying mechanisms assumes paramount importance in devising effective prevention and treatment strategies.^15^ Yet, the precise mechanisms governing irAEs remains to be fully elucidated, and so preventative action has yet to be consistently applied in clinical practice.^15^ Broadly, many irAEs are identified as autoimmune diseases triggered by ICI‐activated CD8+ cytotoxic T cells, and some involve activated B cells and pathogenic antibody production.^16^, ^17^ Additionally, there is growing interest in exploring the involvement of T follicular helper (T_fh_) cells in promoting abnormal B‐cell reactions, potentially disrupting immune tolerance in peripheral tissues.^18^, ^19^ Consequently, there is a pressing need to delve into the underlying mechanisms of ICI‐induced toxicity and formulate mechanism‐based strategies to mitigate its occurrence.^15^ Regrettably, a standardised strategy for patient stratification based on their risk of experiencing toxicity is lacking in the current clinical landscape.^17^ This review endeavours to address these challenges by consolidating the existing knowledge on irAE mechanisms in NSCLC and mitigation strategies, thus serving as a valuable resource for clinicians seeking to enhance irAEs management.

## 2 CLINICAL MANIFESTATION OF ICI‐INDUCED IrAEs IN NSCLC

**FIGURE 1:**
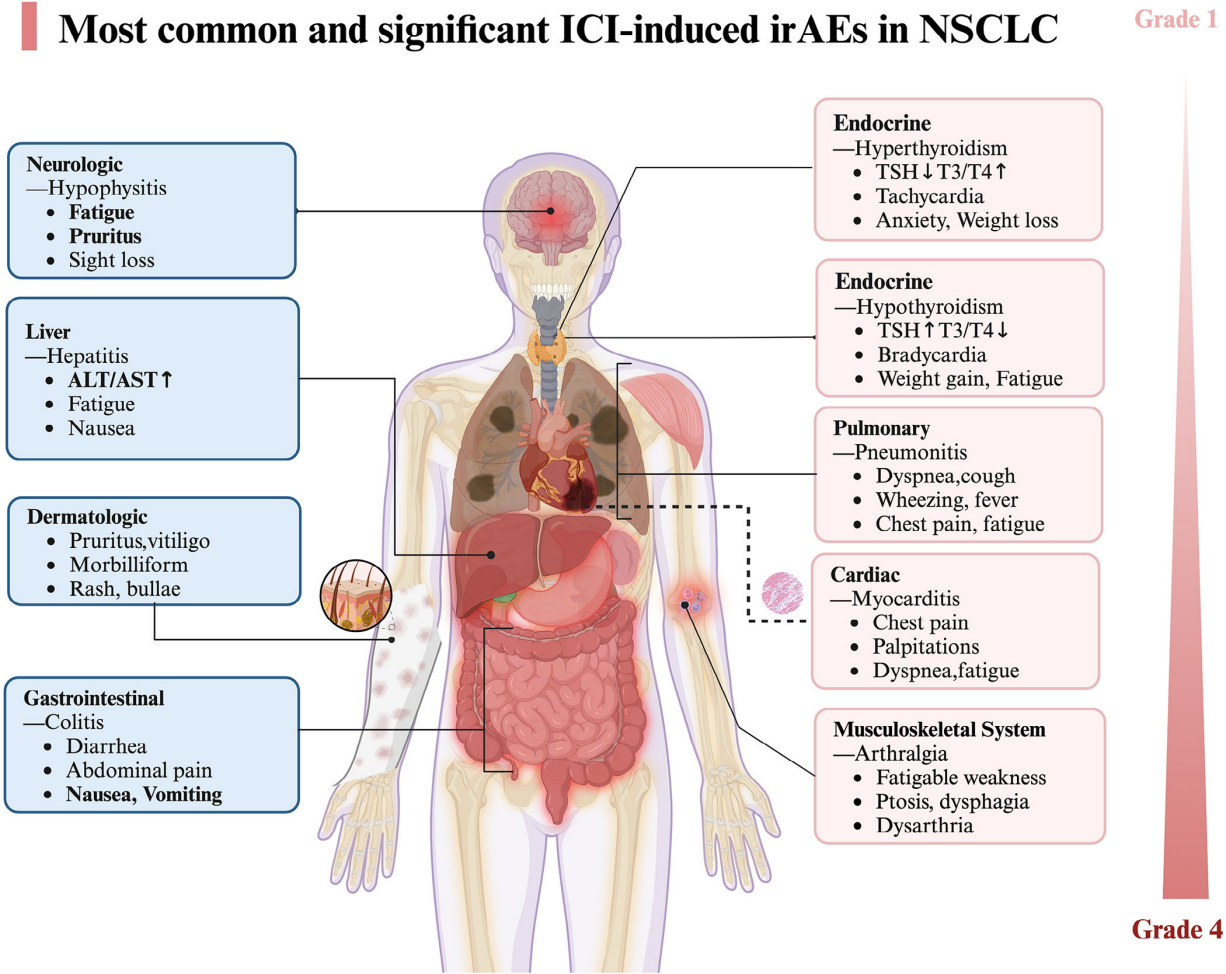
Clinical manifestation and occurrence of immune‐related adverse events (irAEs) in non‐small cell lung cancer (NSCLC).

IrAEs from NSCLC immunotherapy impact various organs, such as the heart, lungs, skin, digestive system, nervous system and muscles (Figure 1).^15^, ^20^, ^21^ A recent systematic review reported that moderate to severe chronic non‐endocrine irAEs could persist for a median (range) of 180 (84–2370) days, with 52% of patients experiencing chronic irAEs that persisted for over 6 months.^20^ Additionally, research has emphasised that the incidence of irAEs within the initial year of anti‐PD‐1 treatment is closely associated with prolonged toxicity extending past 1 year in individuals with advanced NSCLC.^21^ These findings underscore the variability in terms of the occurrence, severity and timing of these toxicities.^20^, ^21^ It is noteworthy that certain acute irAEs occur more frequently with dual immunotherapy than with monotherapy. This suggests that the type and severity of irAEs depend on the affected organ and the specific ICI used^22^ (Figure 1 and Table 1).

**TABLE 1:** Clinical incidence of organ‐specific immune‐related adverse events (irAEs) in non‐small cell lung cancer (NSCLC) patients receiving immune checkpoint inhibitors (ICIs).

	Incidence of any‐grade irAEs (%)			
Specific irAEs	PD‐1 inhibitor	PD‐L1 inhibitor	CTLA‐4 inhibitor	Combination
**Cardiovascular**				
Myocarditis	(a) Pembrolizumab (<1%)^42^, ^43^, ^44^	(a) Atezolizumab (1%–6.7%)^46^, ^47^		(a) IPI + NIVO (≤1%)^62^
**Pulmonary**				
Pneumonia	(a) Cemiplimab (1%–15%)^63^, ^64^, ^65^ (b) NIVO (3.6%–13%)^38^, ^39^, ^66^, ^67^, ^68^ (c)Pembrolizumab (4.4%–23%)^69^, ^70^	(a) Durvalumab (1.6%–16.7%)^31^, ^32^, ^33^ (b) Atezolizumab (5%–30%)^7^, ^34^, ^35^	(a) Tremelimumab (1%–2%)^31^ (b) IPI (2%)^71^	(a) Anti‐TIGIT (tiragolumab) + atezolizumab (2%)^48^ (b) Anti‐TIGIT (vibostolimab) + pembrolizumab (6%)^52^ (c) IPI + NIVO (3%–7%)^5^, ^62^ (d) IPI + pembrolizumab (7%–12.1%)^4^, ^51^
Interstitial lung disease	(a) NIVO (1%–3%)^38^, ^40^	(a) Atezolizumab (1%)^45^	(a) IPI (1%)^71^	(a) IPI + NIVO (1%–2%)^62^, ^72^
**Dermatologic**				
Rash	(a) NIVO (5.7%–27%)^38^, ^39^, ^40^ (b) Pembrolizumab (7%–22%)^42^, ^44^, ^73^ (c) Cemiplimab (5%)^64^	(a) Atezolizumab (5%–11%)^7^, ^35^, ^45^ (b) Durvalumab (3.2%–15%)^31^, ^32^, ^33^	(a) IPI (17%–28%)^71^, ^74^ (b) Tremelimumab (13.3%–41%)^31^, ^75^	(a) Anti‐TIGIT (tiragolumab) + atezolizumab (27%)^48^ (b) IPI + NIVO (10.4%–20%)^49^, ^50^, ^62^, ^72^
**Endocrine**				
Hypo‐thyroidism	(a) NIVO (4%–7.7%)^39^, ^72^ (b) Pembrolizumab (6.7%–28.8%)^42^, ^44^, ^73^, ^76^ (c) Cemiplimab (10%)^41^	(a) Atezolizumab (5%–14.2%)^7^, ^29^, ^45^ (b) Durvalumab (10.5%–15.2%)^32^, ^77^	Tremelimumab (4%)^75^	(a) Anti‐TIGIT (tiragolumab) + atezolizumab (10%)^48^ (b) IPI + NIVO (11%–16%)^49^, ^50^
Hyper‐thyroidism	(a) Pembrolizumab (4%–11.1%)^42^, ^44^, ^73^, ^76^ (b) NIVO (3%–7%)^39^, ^78^	(a) Atezolizumab (2.8%–4.1%)^29^ (b) Durvalumab (6.3%–12.1%)^32^, ^33^, ^77^	(a) IPI (2%)^71^	(a) IPI + NIVO (8.7%)^72^
**Gastrointestinal**				
Nausea	(a) Pembrolizumab (2%–17.2%)^42^, ^44^ (b) NIVO (5%–17%)^79^, ^80^ (c) Cemiplimab (3%)^64^	(a) Atezolizumab (7.7%–14.2%)^11^, ^28^	(a) IPI (8%–18%)^71^, ^81^ (b) Tremelimumab (10%)^31^	(a) Durvalumab + tremelimumab (1.2%–18%)^31^, ^82^
Diarrhoea	(a) Pembrolizumab (48%)^83^ (b) NIVO (8.9%–24%)^46^, ^80^, ^84^ (c) Cemiplimab (5%–24%)^41^, ^64^	(a) Atezolizumab (6.2%–20.6%)^7^, ^11^, ^29^, ^30^ (b) Durvalumab (4%–20%)^31^, ^33^, ^77^, ^85^	(a) IPI (27%–30%)^71^, ^81^	(a) IPI + NIVO (6.4%–20%)^49^
Colitis	(a) NIVO (2%)^38^ (b) Pembrolizumab (1%–3.9%)^44^, ^73^ (c) Cemiplimab (<4%)^64^	(a) Atezolizumab (2.1%)^7^ (b) Durvalumab (1.6%–4%)^31^, ^85^	(a) Tremelimumab (8.8%–19%)^31^, ^75^ (b) IPI (4%)^71^	(a) Anti‐TIGIT (tiragolumab) + atezolizumab (4%)^48^ (b) IPI + NIVO (1%–6%)^62^, ^86^ (c) Durvalumab + tremelimumab (1.8%)^31^
**Liver**				
Hepatitis	(a) Pembrolizumab (2.1%–17.2%)^44^, ^87^ (b) NIVO (2%–10%)^38^, ^39^, ^40^ (c) Cemiplimab (<2%)^64^	(a) Atezolizumab (1%–23%)^28^, ^30^, ^45^ (b) Durvalumab (13%)^33^	(a) IPI (5%–42%)^71^, ^81^ (b) Tremelimumab (8.3%–30%)^31^, ^75^	(a) Anti‐TIGIT (tiragolumab) + atezolizumab (5%)^48^ (b) IPI + NIVO (1%–6%)^50^, ^62^, ^88^
**Renal**				
Nephritis/renal injury	(a) Pembrolizumab (.4%–.6%)^42^, ^73^ (b) Cemiplimab (1%)^41^	(a) Durvalumab (.4%)^77^	(a) IPI (<1%)^71^	(a) IPI + pembrolizumab (1%)^5^ (b) IPI + NIVO (1%)^5^
**Neurologic**				
Encephalitis	(a) NIVO (< 1%)^38^	(a) Avelumab (.3%)^88^		
**Musculoskeletal**				
Arthralgia	(a) Pembrolizumab (4%–20.5%)^44^, ^89^ (b) NIVO (5.7%–26%)^79^, ^80^ (c) Cemiplimab (4%–13%)^41^, ^64^	(a) Atezolizumab (2%–16.8%)^30^, ^45^, ^46^ (b) Durvalumab (16.7%)^32^	(a) IPI (7%–14%)^71^, ^81^	(a) Anti‐TIGIT (tiragolumab) + pembrolizumab (12%)^52^ (b) Anti‐TIGIT (tiragolumab) + atezolizumab (16%)^48^ (c) IPI + pembrolizumab (7%–9.9%)^4^, ^7^
**Eyes**				
Uveitis	(a) Pembrolizumab (.6%)^73^		(a) IPI (1.5%)^90^	(a) IPI + pembrolizumab (9%)^4^

Abbreviations: CTLA‐4, cytotoxic T‐lymphocyte antigen 4; IPI, ipilimumab; NIVO, nivolumab; PD‐1, programmed death‐1; PD‐L1, programmed death‐ligand 1; TIGIT, T‐cell immunoreceptor with immunoglobulin and immunoreceptor tyrosine‐based inhibitory motif domains.

### 2.1 irAEs induced by CTLA‐4, PD‐1 and PD‐L1 inhibitors

The primary categories of ICIs encompass CTLA‐4, PD‐1 and PD‐L1 inhibitors.^23^ CTLA‐4 inhibitors empower T cells to enhance their activity and effectively eliminate malignant cells.^23^, ^24^ Nevertheless, loss of function of the CTLA4 gene, whether induced by genetic knockout in vivo or inhibition in humans, is linked to widespread adverse effects related to autoimmunity across various tissues.^25^ Additionally, considering significant function of PD‐1 in regulating the immune response of activated T cells, targeting PD‐1/PD‐L1 pathway may impede tumour immune evasion, but may also potentially disrupt the balance between normal tissues and cancer cells, resulting in immune imbalance.^23^, ^26^

In NSCLC patients treated with anti‐CTLA‐4, common irAEs include skin rashes (13.3%–41%), liver inflammation (5%–42%) and digestive problems such as diarrhoea (27%–30%), nausea (8%–18%) and colitis (8.8%–19%) (Table 1),^7^, ^11^, ^27^, ^28^, ^29^, ^30^ as depicted in Table 1. However, NSCLC patients on CTLA‐4 inhibitors less frequently develop checkpoint inhibitor pneumonitis (CIP) compared to those on PD‐1/PD‐L1 inhibitors, although underreporting is a concern.^7^, ^31^, ^32^, ^33^, ^34^, ^35^, ^36^ Moreover, research from clinical trials indicates that the range of affected organ systems and the intensity of irAEs differ across therapeutic agents, especially when comparing CTLA‐4 with PD‐1/PD‐L1 inhibitors and their various combinations.^23^ For instance, gastrointestinal toxicity is predominant in NSCLC patients treated with anti‐PD‐1/PD‐L1, with subsequent occurrences of endocrine (6.7%–28.8%), musculoskeletal (5.7%–26%) and pulmonary issues (4.4%–23%)^23^ (Table 1). Furthermore, patients on PD‐1 inhibitors have a notably higher rate of experiencing diarrhoea, skin rashes and kidney damage than those on PD‐L1 inhibitors.^37^, ^38^, ^39^, ^40^, ^41^ Conversely, myocarditis is more common in patients undergoing treatment with anti‐PD‐L1 (1%–6.7%) compared to those on anti‐PD‐1 (<1%)^42^, ^43^, ^44^, ^45^, ^46^, ^47^ (Table 1).

### 2.2 irAEs induced by combination regimens of ICIs

Compared to mono‐immunotherapy or chemotherapy, NSCLC treatment using a combination of ICIs correlates with an increased frequency of irAEs in patients; however, it offers improved survival outcomes^4^. The incidence of endocrine toxicities (8.7%–16%) is elevated in combined immunotherapy compared to monotherapy,^48^, ^49^, ^50^ as shown in Table 1. Additionally, ipilimumab plus pembrolizumab treatment is associated with a higher occurrence of pneumonia (7%–12.1%) compared to pembrolizumab alone^4^, ^51^ (Table 1). Conversely, when PD‐1/PD‐L1 inhibitors are used in combination with other types of ICIs for NSCLC treatment, there is an increased irAEs impacting the skin, digestive system and musculoskeletal system, compared to when PD‐1/PD‐L1 and CTLA‐4 inhibitors are used together^4^, ^7^, ^31^, ^48^, ^52^ (Table 1).

The biological hypotheses explaining how different ICIs cause organ‐specific irAEs are influenced by the affected organ. In the gastrointestinal system, CTLA‐4 is more crucial for regulating gut equilibrium than PD‐1/PD‐L1.^53^ CTLA‐4‐targeting monoclonal antibodies (MoAbs) activate the T cells in the gut, leading to colitis characterised by elevated CD4+ effector and regulatory T cells (Tregs), accompanied by significant changes in Treg gene expression.^54^ Conversely, while PD‐1/PD‐L1 MoAbs also induce gastrointestinal irAEs, they are generally less severe.^53^ Cardiac irAEs stem from disrupted immune balance in the heart, particularly with ICI combinations, with a notable increase in cytotoxic CD8+ T cells and T‐cell receptor (TCR) rearrangement, hint at α‐myosin as a potential driver of myocardial damage.^55^, ^56^ CIP is more likely with PD‐1 inhibitors, which increase cytokine production and CD4+ T‐cell growth compared to CTLA‐4 or PD‐L1 inhibitors, possibly owing to enhanced interaction of repulsive guidance molecule b and PD‐L2 in lung cells.^57^, ^58^ As for dermatologic irAEs, lichenoid dermatitis is often associated with PD‐1/PD‐L1 MoAbs, owing mainly to the activation of CD4+ T cells.^59^ Conversely, alopecia areata is linked to CTLA‐4 gene variants, while CTLA‐4 IgG supplementation can avert its development in mice.^60^ For endocrine‐related irAEs, thyroiditis is often driven by anti‐PD‐1‐activated CD4+ T cells. However, the specific impact of anti‐CTLA‐4 on thyroid function remains unclear.^61^ Therefore, organ‐specific irAEs appear to be influenced by distinct molecular mechanisms of various MoAbs, underscoring the complexity and individual variability of irAEs.

## 3 MECHANISMS OF ICI‐INDUCED irAEs IN NSCLC

**FIGURE 2:**
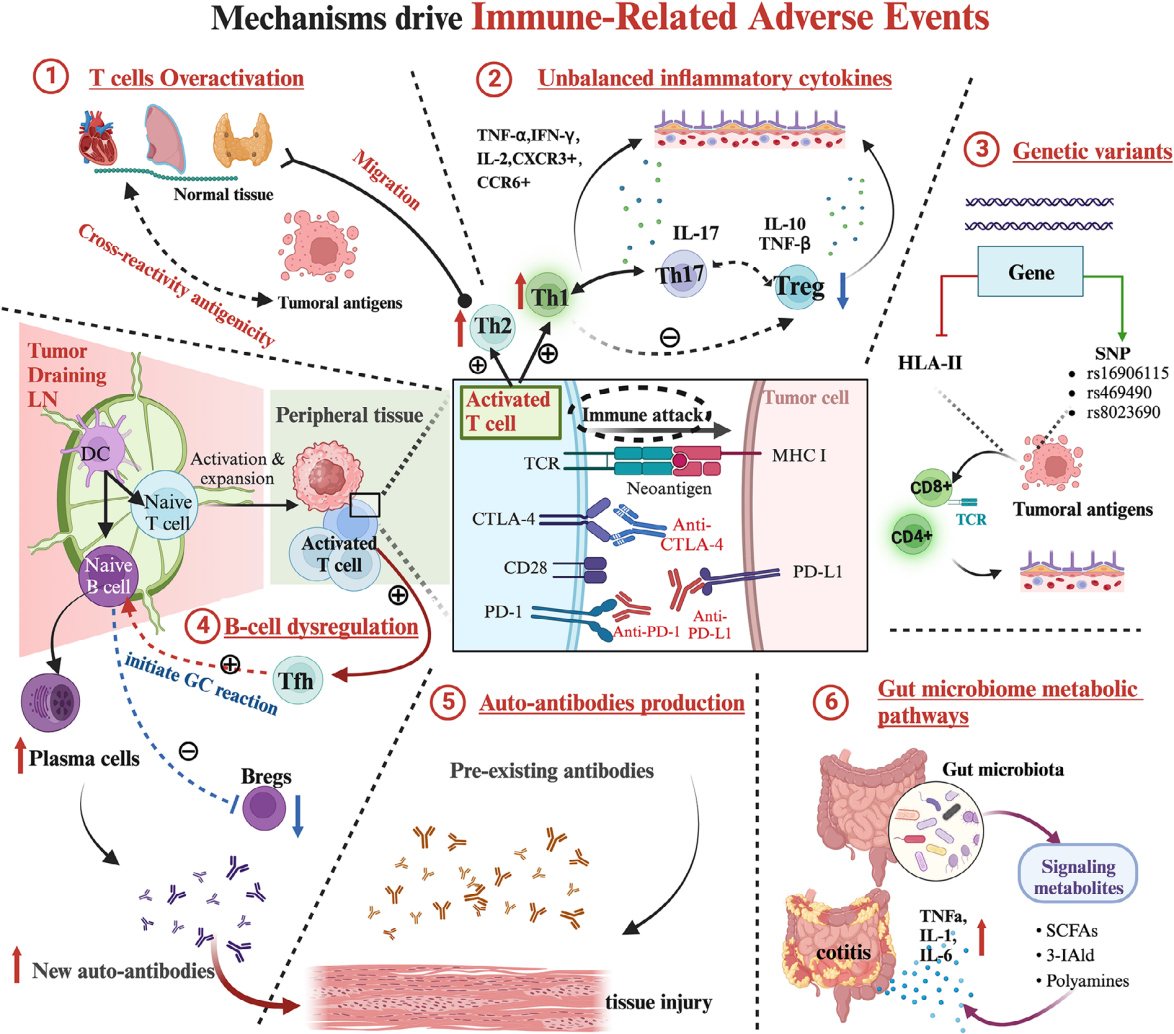
Potential mechanisms involved in the occurrence of immune‐related adverse events (irAEs) in non‐small cell lung cancer (NSCLC). There are multiple potential mechanisms that could contribute to the occurrence of immune checkpoint inhibitor (ICI)‐mediated toxicity. (1) When T cells are activated by ICIs, cross‐reactivity antigenicity can occur between tumours and normal tissue affected by irAEs. Notably, patients who developed irAEs exhibited an increase in proliferative activated T cells in normal tissue. (2) ICIs can induce upregulation in synthesis of inflammatory cytokines and chemokines, and increase in the tissue‐resident populations of T helper cells (Th1/2/17) and decrease in regulatory T cells (Tregs), thereby participating in early development of irAEs. (3) Germline genetic factors including specific human leukocyte antigen (HLA) alleles and single nucleotide polymorphism (SNP) may contribute to irAEs. (4) ICI‐associated increased B‐cell clonality as well as increased detection of autoantibodies of ICIs commencement are associated with irAEs. Additionally, follicular T helper cells (T_fh_) cells typically express programmed death‐1 (PD‐1) and interact with programmed death‐ligand 1 (PD‐L1)‐producing B cells in B‐cell follicle to initiate germinal centre (GC) reaction, whereby B cells undergo maturation. (5) Patients with pre‐existing or new auto‐antibodies may also be an underlying predisposition to the occurrence of irAEs. (6) ICIs can affect the intestinal microbiome profile, leading to production of proinflammatory cytokines.

Currently, accumulating evidence suggests that checkpoint inhibitors could specifically reactivate T cells and self‐antigen‐mediated cellular immunity in normal tissues, which could result in irAEs.^23^, ^67^ Other intrinsic factors could include pre‐existing autoimmunity, genetic variants and unbalanced inflammatory cytokines^91^ (Figure 2). Nevertheless, the underlying mechanisms behind irAEs in NSCLC are still predominantly undetermined.^23^

### 3.1 T‐cell overactivation in the pathogenesis of irAEs

ICIs have been observed to potentially lead to the growth and stimulation of memory T cells in normal tissue in the development of irAEs.^15^, ^23^ Recent research suggests that the infiltration of tissues by active T cells might participate in the formation of irAEs in NSCLC.^15^

#### 3.1.1 Cross‐reactivity antigenicity of T cells between tumoural and healthy cells

The onset of irAEs is believed to be linked to antigen cross‐reactivity between tumoural antigens and their healthy counterparts.^92^ Numerous investigations have empirically validated this observation. For example, active CD8+ T lymphocytes infiltrating healthy cardiac tissue and the consequent T‐cell‐mediated autoimmunity are strongly associated with fulminant myocarditis in melanoma and NSCLC patients receiving anti‐CTLA‐4/PD‐1 therapy, underscoring the pivotal relationship between antigen cross‐presentation and ICI‐induced myocarditis.^36^, ^93^ Additionally, Berner et al.^94^ identified the same TCR sequences in both cancerous and normal tissues in NSCLC patients showing autoimmune skin toxicities post anti‐PD‐1 therapy. Notably, Berner et al.^95^ developed a systematic approach aimed at identifying self‐antigens related to ICI responses and irAEs in NSCLC patients. Remarkably, this study found that napsin A‐specific T‐cell clones were disproportionately represented in ICI responders, lung cancer and post‐therapy inflammatory pulmonary lesions.^95^ This innovative framework paves the way for the identification of irAE‐specific antigens that are potentially targetable.^95^ Historical data further indicate an escalated prevalence of CD8+ T cells in lung samples from NSCLC patients who developed CIP after nivolumab, highlighting the crucial role of antigen cross‐reactivity.^96^ Furthermore, Lechner et al.^97^ found a clonally expanded segment of cytotoxic C‐X‐C motif chemokine receptor 6 (CXCR6)+ CD8+ T cells in thyroid tissue, a population characterised by interferon‐γ (IFN‐γ) and granzyme B upregulation and spurred on by interleukin (IL)‐21, which was indicative of ICI‐induced thyroiditis in NSCLC and other cancer types.^97^ This concept, where T cells recognise tumour‐specific antigens in normal cells leading to irAEs, is reiterated in various studies covering ailments such as vitiligo, colitis and neurological conditions.^94^, ^96^, ^98^, ^99^

Furthermore, ICIs have been demonstrated to enhance the variety of T‐cell clones in both bronchoalveolar lavage fluid (BALF) and circulating blood, leading to the development of irAEs.^94^, ^100^, ^101^ Suresh et al.^100^ performed an examination of lymphocytes from BALF in patients undergoing ICIs therapy. Their findings revealed a notable increase in CD4+ T cells clones among patients with NSCLC with CIP.^100^ Additionally, another study reported an elevated presence of CD8+ T cells with PD‐1, T‐cell immunoreceptor with immunoglobulin and immunoreceptor tyrosine‐based inhibitory motif domains (TIGHIT) and T cell immunoglobulin domain and mucin domain‐3 (Tim‐3) expression in BALF from NSCLC and other cancer patients with PD‐1/PD‐L1 inhibitor‐related interstitial lung disease (ILD), as opposed to other ILD types.^101^ Corroborating this, circulating T cells specific to epidermal antigens were identified in autoimmune skin conditions and lung cancer in NSCLC patients post‐anti‐PD‐1 treatment, who showed skin‐related adverse effects.^94^ This revelation enhances our comprehension of circulating antigen cross‐presentation and its influence on organ‐specific irAEs in NSCLC.^94^ Recent findings also postulate that activated CD4+ T cells can induce inflammatory cell death, thereby managing immune‐evasive tumours.^102^ The concurrent presence of activated baseline CD4 memory T‐cell clones and varied TCR, as mapped through single‐cell RNA sequencing (scRNA‐seq), is associated with the development of irAEs in melanoma patients, regardless of the specific organ systems implicated.^103^ While such observations have yet to be noted in circulating blood samples from NSCLC patients, future scRNA‐seq investigations into NSCLC could potentially illuminate the indispensable role of T‐cell clones in precipitating irAEs.

#### 3.1.2 Activation of T helper cells and Tregs involved in irAEs

Within the myeloid compartment, dendritic cells and macrophages are crucial for presenting antigens, which in turn helps activate T cells.^18^ Following activation by myeloid cells in tumour‐draining lymph nodes (dLNs), CD4+ and CD8+ T cells differentiate into various tumour‐infiltrating lymphocytes (TILs), encompassing T helper (Th1/Th2/Th17) cells, Tregs and T_fh_ cells (CD4+ CXCR5+ PD‐1^high^), among others.^18^ Post‐differentiation, these TILs exit dLNs, migrate to peripheral tissues and instigate a nascent antibody response.^18^ Notably, these TILs, while instrumental in the anti‐tumour immune response, can manifest divergent roles in irAEs.^18^

It has been suggested that anti‐PD‐1/PD‐L1 treatments bolster Th1 cell proliferation while concurrently attenuating Th2‐associated cytokine production, which collectively inhibits tumour growth.^15^, ^23^ Interestingly, it was observed that CD8+ Th1 cells expressing CX3CR1/CXCR3 were significantly expanded in arthritis irAEs among NSCLC and other cancer patients, implicating Th1 cells in irAE pathogenesis.^104^ Subsequent research has shown that anti‐PD‐1/PD‐L1 can hinder the transformation of Th1 cells into Tregs, potentially exacerbating immune‐mediated damage.^105^ Suresh et al.^100^ observed a reduction in CTLA‐4+ and PD‐1+ Tregs in the BALF of CIP patients, suggesting a possible involvement of Tregs in the formation of irAEs. Despite their capacity to inhibit Th1 cell growth, Tregs fail to prevent the transformation of Th17 cells into Th1 cells.^106^ Interfering with the PD‐1/PD‐L1 pathway leads to a decrease in Tregs, potentially leading to dysregulation of the Treg/Th17 cell balance, which has been implicated in various autoimmune disorders.^92^ Recent scRNA‐seq studies involving patients with NSCLC have observed an elevation in CD4+ Th2 cells and CD4+ Th17 cells linked to pneumonitis and thyroiditis, respectively, highlighting their proinflammatory effects and the potential occurrence of organ‐specific toxic effects.^107^ Moreover, the myeloid compartment is vital in the pathogenesis of ICI‐mediated irAEs, acting as essential antigen‐presenting cells for the activation of T cell and significantly influencing PD‐1 expression beyond T cells.^108^, ^109^ Anti‐PD‐1 therapy can induce alterations within the myeloid compartment, leading to a systemic accumulation of inflammatory cells across various organs and tissues.^110^, ^111^ This has been particularly demonstrated by the expansion of inflammatory macrophages in mouse models of ICI‐associated myocarditis.^110^, ^111^ Moreover, the role of macrophages in the onset of ICI‐induced diabetes underscores their substantial influence in the occurrence of irAEs, thus highlighting the extensive effects of myeloid cells in ICI‐related adverse effects.^112^

#### 3.1.3 T follicular helper cells involved in irAEs

It is crucial to acknowledge the capacity of activated CD4+ T cells to differentiate into early T_fh_ cells. These early T_fh_ cells subsequently engage in intricate interactions with antigen‐specific B cells and migrate into the follicle, where they establish the germinal centre (GC) and initiate the early extrafollicular antibody response.^18^, ^19^ These T_fh_ cells display increased expression of costimulatory receptors including inducible T cell co‐stimulator (ICOS), as well as coinhibitory receptors (such as PD‐1) on their cell surface, while also secreting IL‐21 and IL‐4, thus participating in potential pathways that are involved in the regulation of humoral immunity.^18^, ^113^ Consequently, as has been previously hypothesised, PD‐1 inhibitors could potentially disrupt T_fh_ cell function, resulting in the generation of aberrant B cells and ultimately leading to the occurrence of irAEs.^18^, ^114^ More recently, Lechner et al.^97^ identified the infiltration of both T peripheral helper (T_ph_) and T_fh_ cells into ICI‐related thyroiditis tissue in patients with NSCLC and other types of tumours. They proposed a potential mechanism in vivo in which IL‐21+ T_fh_ and T_ph_ cells might significantly contribute to ICI‐induced autoimmunity by augmenting the effector characteristics of CD8+ T cells.^97^ Specifically, recombinant IL‐21 has been shown to activate CD8+ effectors expressing CXCR6, GZMB and IFN‐γ, thereby enhancing thyrotoxic activity in mice. This suggests that targeting IL‐21 signalling could potentially reduce irAEs.^97^ Notably, the observed pattern of irAEs in the mouse model may not fully correspond to that observed in human patients. Thus, future endeavours should amalgamate insights from both patient‐derived irAE data and syngeneic murine tumour models to elucidate the nuanced interplay between IL‐21‐mediated autoimmunity and antitumour responses.

### 3.2 Unbalanced inflammatory cytokines involved in irAEs

**FIGURE 3:**
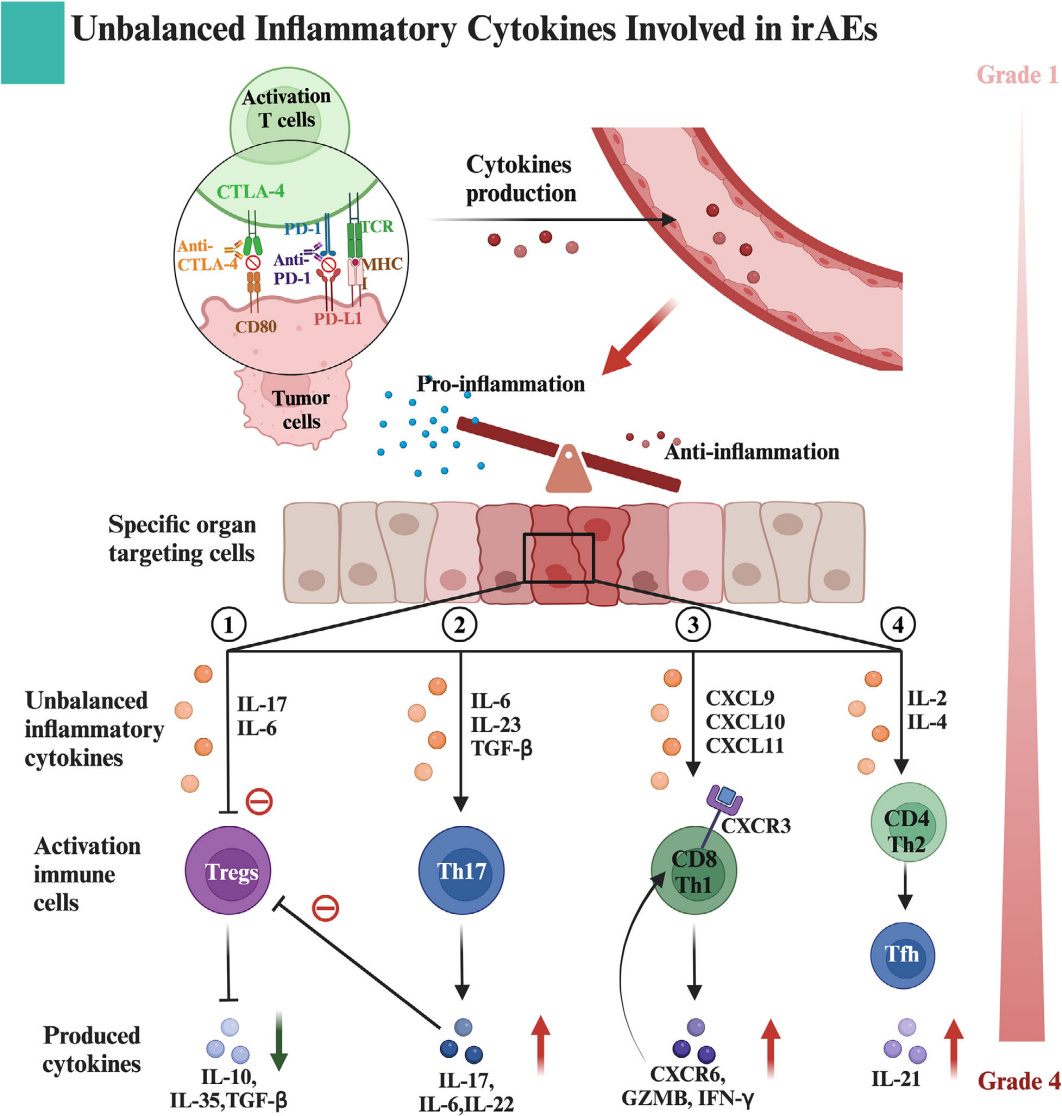
Unbalanced inflammatory cytokines in immune‐related adverse events (irAEs). Immunotherapy may lead to cytokine dysregulation, causing systemic inflammatory responses that further promote organ‐specific irAEs. (1) Circulating interleukin (IL)‐17 and IL‐6 secreted by T helper 17 cells (Th17) cells could inhibit the activity of regulatory T cells (Tregs), thereby reducing the production of anti‐inflammatory factors such as IL‐10, IL‐35 and transforming growth factor‐beta (TGF‐β), which are associated with the occurrence of irAEs. (2) Cytokines like IL‐6, IL‐23 and TGF‐β stimulate Th17 to secrete IL‐17 and IL‐6, contributing to irAEs. (3) C‐X‐C motif chemokine ligand 9/10/11 (CXCL9/10/11) binds to C‐X‐C motif chemokine receptor 3 (CXCR3) on cytotoxic T cells, stimulating their activation and promoting CXCR6, granzyme B (GZMB) and interferon‐γ (IFN‐γ) secretion. This leads to the progression of irAEs in non‐small cell lung cancer (NSCLC). (4) Other cytokines such as IL‐2 and IL‐4 stimulate CD4+Th2 cells to secrete proinflammatory factors, including IL‐21.

Multiple studies have indicated that the dysregulation of cytokine secretion serves as an additional catalyst for the development of irAEs.^91^ Cytokines are critical in regulating immune system functions and supporting the activities of different immune cells.^91^ They regulate a variety of signalling pathways essential for T‐cell activation and the transformation of B cells into plasma cells, which are responsible for antibody production.^91^ The presence of proinflammatory cytokines has been shown to initiate a systemic inflammatory response, thereby increasing the likelihood of irAEs^115^ (Figure 3). Notably, elevated levels of circulating IL‐17 and IL‐6, secreted primarily by Th17 cells that suppress Treg activity, along with IL‐2, known for boosting cytotoxic CD8+ T‐cell activity, are associated with irAEs onset.^91^, ^115^, ^116^, ^117^ Lim et al.^115^ observed an association between the levels of 11 cytokines including Fractalkine, granulocyte colony stimulating factor (G‐CSF), fibroblast growth factor 2 (FGF‐2), granulocyte‐macrophage colony‐stimulating factor (GM‐CSF), IFN‐α2, IL‐1a, IL‐1b, IL‐1RA, IL‐2, IL‐12p70 and IL‐13 in the blood and the emergence of irAEs in melanoma patients receiving anti‐PD‐1 treatment. This research also created a toxicity index using these 11 cytokines to identify patients at increased risk of experiencing irAEs.^115^ Chen et al.^118^ conducted scRNA‐seq on lung adenocarcinoma patients undergoing ICI treatments, wherein they observed a notable elevation in the levels of circulating tumour necrosis factor (TNF) protein among patients experiencing irAEs, a trend not observed in those responding to ICI treatments.^118^ Another study, primarily involving NSCLC patients, showed a notable link between C‐X‐C motif chemokine ligands (CXCLs) and irAEs incidence. Specifically, CXCL9/10/11 were identified as binding to CXCR3, thereby stimulating T‐cell activation and contributing to the progression of irAEs in NSCLC.^119^

Regarding organ‐specific irAEs, Lechner et al.^97^ discovered IL‐21, secreted by CD4+ T_fh_ and T_ph_ cells, significantly contributes to the emergence of both ICI‐induced thyroiditis and Hashimoto's thyroiditis in NSCLC patients. Consequently, this finding could elucidate why patients with existing thyroid autoantibodies are more prone to developing thyroid irAEs when undergoing ICI treatments.^120^ Furthermore, various research has shown that increased levels of serum inflammatory indicators such as IL‐6, IL‐10, IL‐17, IL‐35 and C‐reactive protein correlate with a heightened risk of severe irAEs in NSCLC.^121^, ^122^, ^123^ Additionally, apart from being present in tissue and serum, certain proinflammatory and chemotactic cytokines linked to CIP are detectable in the BALF of NSCLC patients.^100^, ^122^ Together, these studies highlight the crucial role of cytokines in the manifestation of irAEs in NSCLC.

### 3.3 Genetic variants associated with irAEs development

Germline genetic factors exert significant influence on immune homeostasis and our immunological status.^17^, ^124^ The variability in the onset and severity of irAEs observed among patients receiving similar ICI agents can be partially attributed to germline genetic variation in immune function.^17^, ^124^ Recent research has demonstrated a correlation between polygenic germline risk for autoimmune conditions and the development of cutaneous and thyroid irAEs in NSCLC.^125^, ^126^ These results supported that germline genetic factors may contribute to the occurrence of irAEs. However, the extent to which individual genetic variants are associated with irAEs in NSCLC continues to be uncertain. For instance, a study by Groha et al.^127^ used a genome‐wide association approach to investigate irAEs, revealing a significant association between a single nucleotide polymorphism known as rs16906115, located within the IL7 gene, and the development of any grade of irAE toxicities.^127^ This correlation was noted in 1751 patients with 12 distinct types of cancer, NSCLC included, all of whom received anti‐PD1/PD‐L1 treatment.^127^ Notably, rs16906115 represents the first genetic variant to be identified as being associated with irAEs using large sample sizes and validation cohorts.^127^ This study primarily focused on patients who were administered PD1/PD‐L1 treatment as single medications. Additionally, another pan‐tumour genome‐wide association study investigating irAEs has identified rs469490 as being associated with any grade of irAEs induced by nivolumab, and rs8023690 as potentially predictive of hypothyroidism in subgroup analysis.^128^ However, it is still unknown whether specific genetic variants are also linked to anti‐CTLA‐4‐related irAEs in NSCLC.

Human leukocyte antigen class II (HLA‐II) is vital in presenting tumour antigens to CD8+ and CD4+ T cells, thereby aiding in the successful eradication of cancer cells.^129^ Interestingly, recent research has shown that genotyping of HLA antigens is a contributing factor in the emergence of particular irAEs.^130^ For example, in a novel prospective study, the links between HLA‐DRB111:01 and pruritus, as well as HLA‐DQB103:01 and colitis, were explored in NSCLC and melanoma patients undergoing ICI therapy.^130^ Additionally, other more focused studies have investigated the connections between particular irAEs and HLA variants in a broad tumour spectrum, including NSCLC, such as HLA‐DR15 and hypophysitis,^131^ HLA‐DRB1 and arthritis,^132^ and HLA‐DR11 and pneumonitis.^133^ These results bolster the theory that genetic variations involved in immune regulation might significantly influence the risk of irAEs in NSCLC. Further investigation through association studies is necessary to enhance the utilisation of genetic factors involved in irAEs in NSCLC.

### 3.4 B‐cell dysregulation in ICI‐induced toxicity

Emerging evidence increasingly underscores the pivotal role of B‐cell dysregulation in mediating both the effectiveness of ICIs and the frequency of irAEs.^134^ However, the exact mechanism by which B cells modulate or mediate severe irAEs remains elusive in NSCLC.^134^ B‐regulatory cells (Bregs) are important immune modulators, preventing excessive inflammation and maintaining immune homeostasis via cytokine production, including IL‐10, IL‐35 and transforming growth factor beta.^18^ Rosser et al.^135^ have identified a decrease in the number and function of Bregs in various irAEs, including autoimmune diseases, chronic infections and cancers.^135^ More recently, subsequent research from Patel et al.^136^ showed a correlation between IL‐10 produced by Bregs and the occurrence of severe irAEs in NSCLC patients treated with ICIs.

Nevertheless, certain Breg subsets, such as the PD‐L1^high^ Bregs, can suppress immune responses independently of IL‐10.^113^ These PD‐L1^high^ Bregs have been described as suppressing B cell subsets via PD‐L1 independently of IL‐10 by impeding T_fh_ cell differentiation, thereby inhibiting the T‐cell response.^113^ T_fh_ cells commonly exhibit PD‐1 and engage with PD‐L1‐expressing B cells in the B‐cell follicle, instigating the GC reaction, leading to B‐cell maturation (Figure 2). Previous evidence has shown that increased presence of T_fh_ cells and PD‐L1^high^ Bregs is a notable feature in autoimmune disorders, especially rheumatic diseases, resulting in heightened proliferation and cytokine output by CD8+ T cells.^18^, ^137^ In a recent longitudinal analysis, four phenotypes of PD‐L1^high^ Breg populations were notably prevalent in non‐toxic NSCLC patients treated with anti‐PD‐1/PD‐L1 through functional ex vivo and deep phenotyping mass cytometric assays.^135^ These findings suggest that NSCLC patients possessing inherent functional impairments in their Breg array and lacking certain peripheral Breg phenotypes may be more inclined to experience severe autoimmune reactions.

### 3.5 Autoantibody production and pre‐existing autoantibodies

An expanding range of studies indicates that the emergence of irAEs in NSCLC may be associated with the presence of both pre‐existing and new autoantibodies in immune responses.^92^ The potential mechanism by which ICIs facilitate the production of autoantibodies could entail interactions between B and T_fh_ cells. The disruption of B‐cell homeostasis, particularly Bregs, by ICIs can lead to autoantibody generation, as evidenced in PD‐1 knockout mice and ICI‐treated patients.^92^ An increase in circulating plasmablasts and associated irAEs has been observed in patients receiving anti‐CTLA‐4 or combination therapies.^92^, ^138^ DeFalco et al.^138^ provided evidence of somatic hypermutation and clonal expansion in these plasmablasts through B cell receptor sequencing analysis. This indicates a strong connection between B‐cell dysregulation and autoantibody production leading to irAEs. Furthermore, ICIs may disrupt T_fh_ cells, which are vital for B‐cell maturation and antibody production, implying that T_fh_ cells contribute to autoantibody production.^18^, ^114^ Therefore, ICIs may directly contribute to autoantibody production by directly affecting B cells and indirectly by altering T_fh_ cell function.

Furthermore, it is increasingly evident that autoantibodies can induce irAEs by inducing systemic inflammation, which subsequently results in tissue damage.^139^, ^140^, ^141^ For example, in a retrospective study of 137 NSCLC patients treated with anti‐PD‐1, a notable association was found between pre‐existing antibodies such as rheumatoid factor, antinuclear antibody and anti‐thyroglobulin, and the incidence of irAEs.^141^ In addition, Osorio et al.^142^ discovered a correlation between ICI‐thyroiditis and anti‐thyroid antibodies in NSCLC. These findings suggest that the presence of heightened levels of pre‐existing or new autoantibodies contributes to irAEs in NSCLC.

### 3.6 Gut microbiome metabolic pathways associated with irAEs

Emerging evidence suggests that specific gut microbiota members, such as Faecalibacterium, Bacteroides and Clostridium, contribute to maintaining immune balance in the host. This balancing act is achieved through the promotion of Treg growth and cytokine production, factors implicated in the onset of irAEs.^143^ Short‐chain fatty acids (SCFAs), including propionate, butyrate, acetate and valeric acid, are essential in engaging with immune cells, particularly colonic macrophages, and suppressing the synthesis of proinflammatory cytokines such as IL‐1, TNF‐α and IL‐6.^144^, ^145^, ^146^ Similar to SCFAs, compounds such as indole‐3‐carboxaldehyde and polyamines, including spermine, are expected to offer defense against the development of irAEs.^143^, ^147^ Particularly, a study of the stool microbiota in 26 patients showed that most baseline phylotypes in those with colitis belonged to the Firmicutes group, whereas individuals without colitis fell under the Bacteroidetes category.^148^ This observation was corroborated by another investigation involving 34 patients, indicating an inverse relationship between the prevalence of the Bacteroidetes phylum and the onset of CTLA4‐related colitis.^139^ More recently, Hamada et al.^149^ observed a higher abundance of Turicibacter and Acidaminococcus in the group of patients experiencing irAEs. These findings collectively imply a potential mechanistic association between particular bacterial groups in the gut microbiota and irAEs, offering a promising target for novel anti‐tumour immunotherapy.

### 3.7 Emerging biomarkers for predicting irAEs

Accumulating data have underscored the critical role of molecular pathological epidemiology in understanding the interrelationships between host exposures, tumour molecular characteristics and host immunity in immunotherapy.^150^, ^151^ External exposures, such as smoking, diet and lifestyle, alongside intrinsic host factors such as the tumour microenvironment, host genomics and systemic factors (e.g., gut microbiota), significantly affect the therapeutic response to ICIs and may possibly lead to irAEs.^145^, ^152^, ^153^, ^154^ In‐depth studies on these relationships have contributed to a more comprehensive understanding of how individual modifiable exposure factors affect the occurrence of irAEs.^150^ For example, patients with a smoking history are more susceptible to the development of CIP during ICI treatment.^152^ Although obesity may be associated with improved responses and survival rates in patients with NSCLC and other solid tumours treated with ICIs,^155^, ^156^ notably, a retrospective study on NSCLC patients receiving anti‐PD‐1/PD‐L1 therapy showed a positive correlation between increased body mass index (BMI) and irAEs risk.^153^ Leiter et al.^157^ also found that overweight patients (BMI ≥25 kg/m^2^) with fewer metabolic complications had a higher risk of irAEs occurrence. These studies highlight the value of lifestyle factors such as diet and smoking in assessing irAE risk. As previously mentioned, the role of the gut microbiome is vital in maintaining homeostasis in the host's gut and other sites, significantly influencing the development of irAEs.^145^, ^154^ Consequently, the role of specific gut microbiomes is gaining increasing recognition.^139^, ^143^, ^149^ Future research should focus on identifying specific microbiomes that can optimise the outcomes of cancer immunotherapy. Therefore, considering the heterogenous nature of irAEs, future research should comprehensively integrate and analyse data on exposure factors, microbiomes, immune status, etc.^150^, ^151^ This integrated approach will help optimise immunotherapy strategies, minimise adverse reaction risks and enhance treatment efficacy.

## 4 MANAGEMENT STRATEGIES TO ABROGATE irAEs OF ICIS IN NSCLC

**FIGURE 4:**
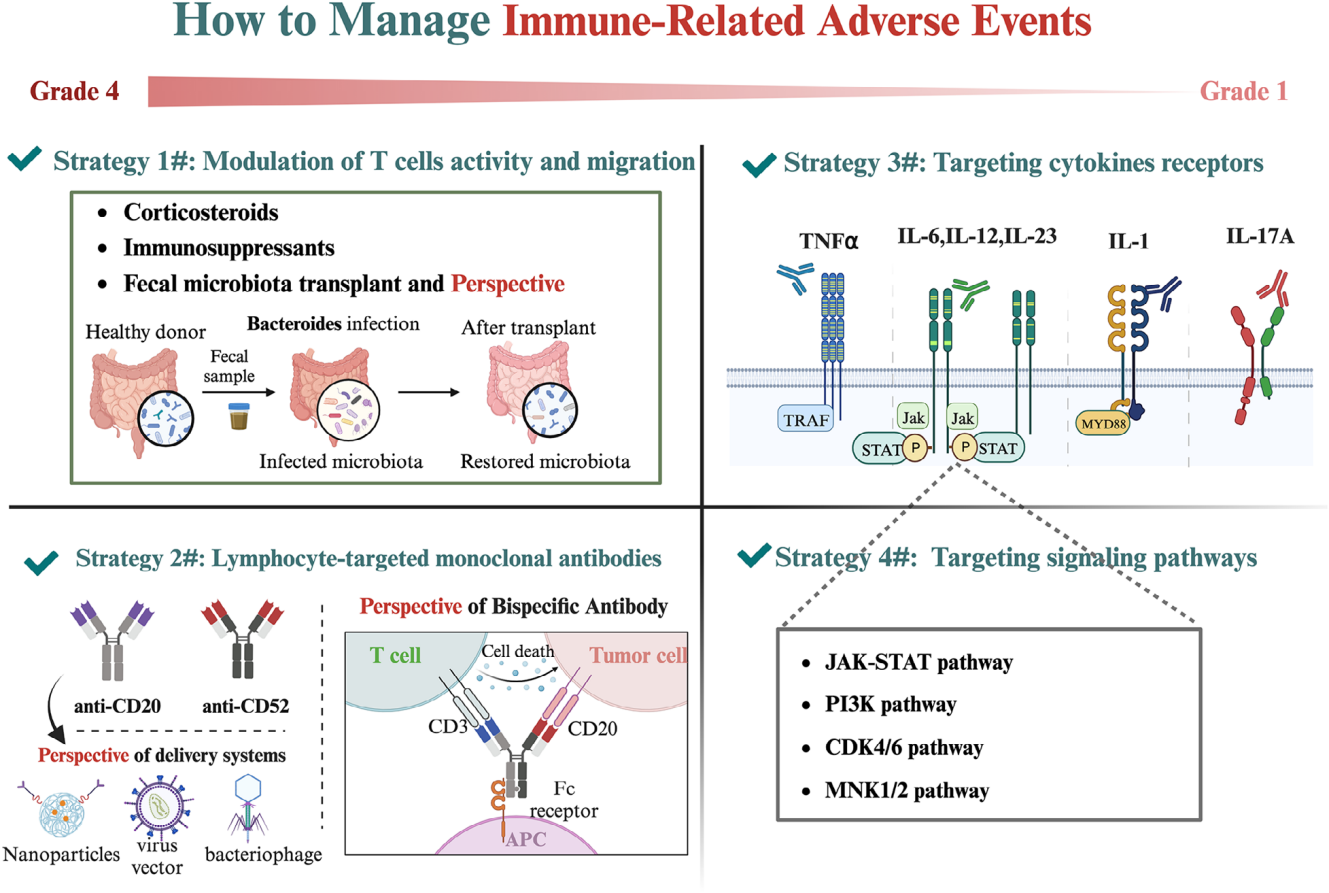
How to manage immune‐related adverse events. Mechanism‐based therapeutic managements for the management of immune‐related adverse events (irAEs), including modulation of T cells activity and migration, monoclonal antibodies, targeting of cytokines receptors and signalling pathways are demonstrated.

**FIGURE 5:**
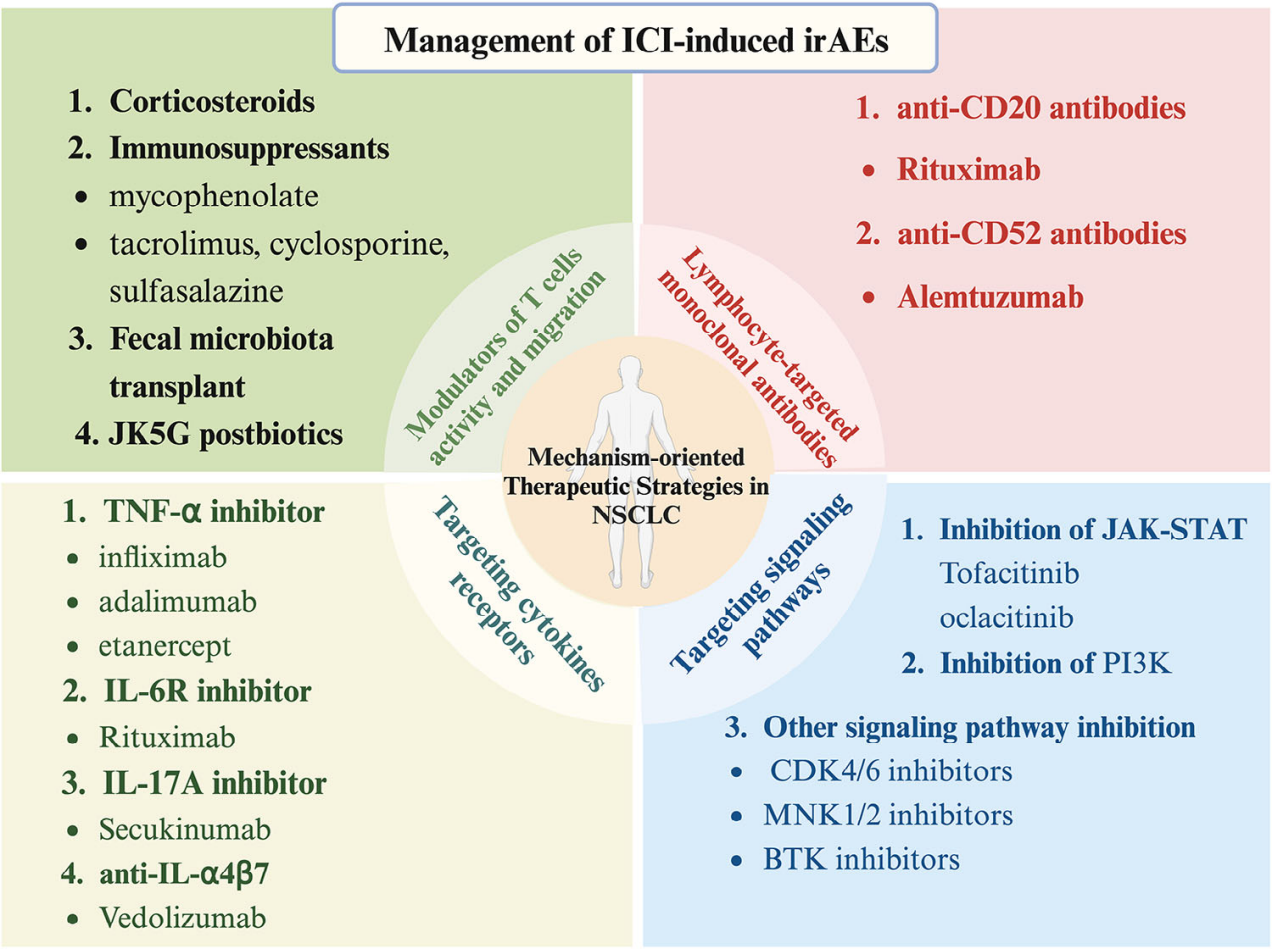
Summary of therapeutic drugs targeting different mechanisms for the management of immune‐related adverse events (irAEs). Therapeutic drugs targeting T cells activity and migration, monoclonal antibodies, targeting of cytokines receptors and signalling pathways are included.

The effective management of irAEs holds significance for patients with NSCLC owing to their propensity to affect various organ systems and potentially lead to fatal outcomes.^15^, ^67^ In light of the increasing understanding of the multifaceted mechanisms underlying irAEs, an array of therapeutic approaches has emerged to address the pathogenesis of these events and mitigate their adverse effects.^15^ Beyond the conventional use of corticosteroids, current research efforts are concentrated on the development of targeted therapeutic strategies tailored to alleviate irAEs.^158^ These strategies encompass the modulation of the T‐cell response and migration, implementation of MoAbs, targeting of secreting cytokines, and inhibition of signalling pathways (Figures 4 and 5).

### 4.1 Modulation of T‐cell activity and migration

Given that T‐cell activation or reactivation is commonly recognised as a key factor in the development of ICI‐related irAEs,^107^ targeting T‐cell activity and movement might serve as an efficient approach to control irAEs in NSCLC (Figures 4 and 5).

#### 4.1.1 Corticosteroid treatment

Corticosteroids have wide‐ranging impacts on multiple immune cells, such as boosting Treg cell production and activity, hindering TCR signalling, diminishing T‐cell effector abilities and favouring a proinflammatory cytokine milieu. Additionally, corticosteroids enhance the expression of immune checkpoints such as PD‐1, CTLA‐4, TIM‐3 and lymphocyte‐activation gene 3 (LAG‐3) on T cells in NSCLC and various other cancers.^27^, ^159^, ^160^, ^161^ Consistent with established management guidelines, glucocorticoids are recommended as the initial therapeutic approach for moderate to severe irAEs in NSCLC.^162^, ^163^, ^164^ Nevertheless, there are conflicting reports concerning the effects of simultaneous corticosteroid and ICI treatment on tumour progression and patient survival.^161^, ^165^, ^166^, ^167^ For instance, Skribek et al. revealed that glucocorticoids effectively mitigated mild irAEs without hindering the effectiveness of ICI therapy in NSCLC patients,^168^ while other studies have shown that high‐dose corticosteroids have a detrimental impact on the effectiveness of ICIs.^23^ Hence, there is still ambiguity regarding whether the observed survival disadvantage can be solely attributed to the administration of high‐dose corticosteroids or overall aggressive immunosuppression.

#### 4.1.2 Immunosuppressants

Furthermore, the inclusion of synthetic immunosuppressive agents as adjuncts to glucocorticoids is imperative in managing steroid‐resistant irAEs by obstructing T‐cell movement, growth or reactivation in NSCLC.^169^ For instance, individuals experiencing steroid‐refractory pneumonitis, hepatitis, nephritis, pancreatitis and uveitis may benefit from treatment with immunosuppressants, such as hydroxychloroquine and mycophenolate, in NSCLC and other cancer types.^169^, ^170^ Less commonly used immunosuppressive therapies for steroid‐refractory irAEs encompass cyclosporine, tacrolimus and sulphasalazine.^169^ The use of these drugs should be contemplated solely for irAEs that are unresponsive to corticosteroids, in conjunction with consultation from relevant specialists in the respective disease field.

#### 4.1.3 Faecal microbiota transplantation and modulation

Recent research has introduced new findings indicating that altering the gut microbiome via faecal microbiota transplantation can efficiently relieve colitis linked to ICIs while reducing side effects from corticosteroids or immunosuppressives.^171^ This approach has been found to significantly reduce the density of CD8+ T cells and increase Treg numbers, providing a potential target for new anti‐cancer treatments.^171^ A recent forward‐looking clinical trial demonstrated that a microecological preparation (JK5G) corresponded with fewer irAEs compared to the control group in NSCLC patients undergoing ICIs and chemotherapy.^172^ Importantly, JK5G postbiotics may also enhance the composition of the gut microbiota and positively modify the tumour milieu by elevating the levels of circulating CD3+CD4+ T cells and CD4/CD8 ratio.^172^

### 4.2 Lymphocyte‐targeted monoclonal antibodies

Currently, the utilisation of lymphocyte‐targeted MoAbs is emerging as a pivotal innovation, particularly for managing severe steroid‐refractory irAEs in NSCLC.^169^, ^173^ MoAbs, such as rituximab, which targets CD20, and alemtuzumab, which targets CD52, have exhibited efficacy in this respect.^169^, ^173^ For instance, Santoro et al.^174^ indicated the successful efficacy of rituximab in treating a patient with lung carcinoid tumours with steroid‐refractory pancreatitis induced by atezolizumab. Similarly, alemtuzumab, which primarily targets the CD52 antigen on B and T lymphocytes, has demonstrated efficacy in treating ICI‐related myocarditis in NSCLC and melanoma patients.^175^ However, management decisions are mostly based on case reports, because prospective or comparative data on the outcome of those MoAbs are lacking. Some prospective studies are underway investigating the efficacy of rituximab/tocilizumab (NCT04375228) and CD24Fc (NCT04552704) in steroid‐refractory irAEs in solid tumours including NSCLC. In recent years, bispecific antibodies have shown strong efficacy in the treatment of malignant tumours, including NSCLC.^176^, ^177^ Double antibodies targeting two independent epitopes or antigens have undergone evaluation in the transformation and clinical research for NSCLC.^176^, ^177^ It is expected that these drugs will lead to a breakthrough in lung cancer treatment.

### 4.3 Targeting cytokines and their receptors

In contrast to corticosteroids that generally suppress various inflammatory pathways, cytokine inhibitors offer a more precise clinical method to diminish inflammation caused by ICIs in NSCLC.^91^ Cytokine inhibitors, including anti‐TNF‐α agents (infliximab, etanercept, adalimumab) and anti‐IL‐17A agents (secukinumab) inhibitors, as well as cytokine receptor inhibitors, such as IL‐6 (tocilizumab) or IL‐α4β7 receptor (vedolizumab) inhibitors, have been investigated for their efficacy in managing steroid‐resistant irAEs in NSCLC.^91^, ^158^, ^178^ Notably, TNFα inhibition has shown effectiveness in treating severe and refractory irAEs, such as colitis, inflammatory arthritis and hepatitis, in NSCLC and other cancer types.^179^, ^180^, ^181^ A recent multicentre study found that inhibition of the IL‐6 receptor improved irAEs in 73% of patients with NSCLC and other cancers without affecting tumour immunity.^178^ Additionally, tocilizumab was notably effective in treating nivolumab‐induced pneumonitis, with a 79.4% positive response in patients with NSCLC.^158^ Moreover, a multicentre study revealed that infliximab or vedolizumab has been shown to reduce severe ICI‐related enterocolitis recurrence.^182^ These studies provide substantial support for ongoing clinical trials assessing the safety and effectiveness of combining tocilizumab and vedolizumab with ICIs in NSCLC (NCT04691817, NCT04940299 and NCT04407247). Furthermore, IL‐17 inhibitors have shown profound therapeutic potential in addressing irAEs, such as intestinal issues, arthropathy and psoriasis, in NSCLC, although their use may potentially promote tumour immune escape.^183^, ^184^ Further clinical trials are underway in NSCLC to assess the effectiveness of tocilizumab in managing migrating irAEs (NCT04691817 NCT04940299). Despite the limited research data on the use of cytokine inhibitors for irAEs, the advancement of specialised cytokine treatments remains promising.

However, the use of anti‐TNF agents is under scrutiny for its potential adverse impact on survival outcomes in immunotherapy. For example, van Not et al.,^185^ in a 2022 JAMA Oncology study, reported reduced OS among patients with NSCLC who received anti‐TNF agents or other immunosuppressants along with steroids. Verheijden et al.^186^ also discovered that the use of anti‐TNF agents might adversely impact OS in patients treated with steroid‐resistant ipilimumab and anti‐PD‐1. Moreover, the increased risk of opportunistic infections associated with anti‐TNF agent administration is becoming increasingly concerning, highlighting the need for careful use of such immunosuppressive treatments in managing irAEs.^187^, ^188^

### 4.4 Targeting signalling pathways

**FIGURE 6:**
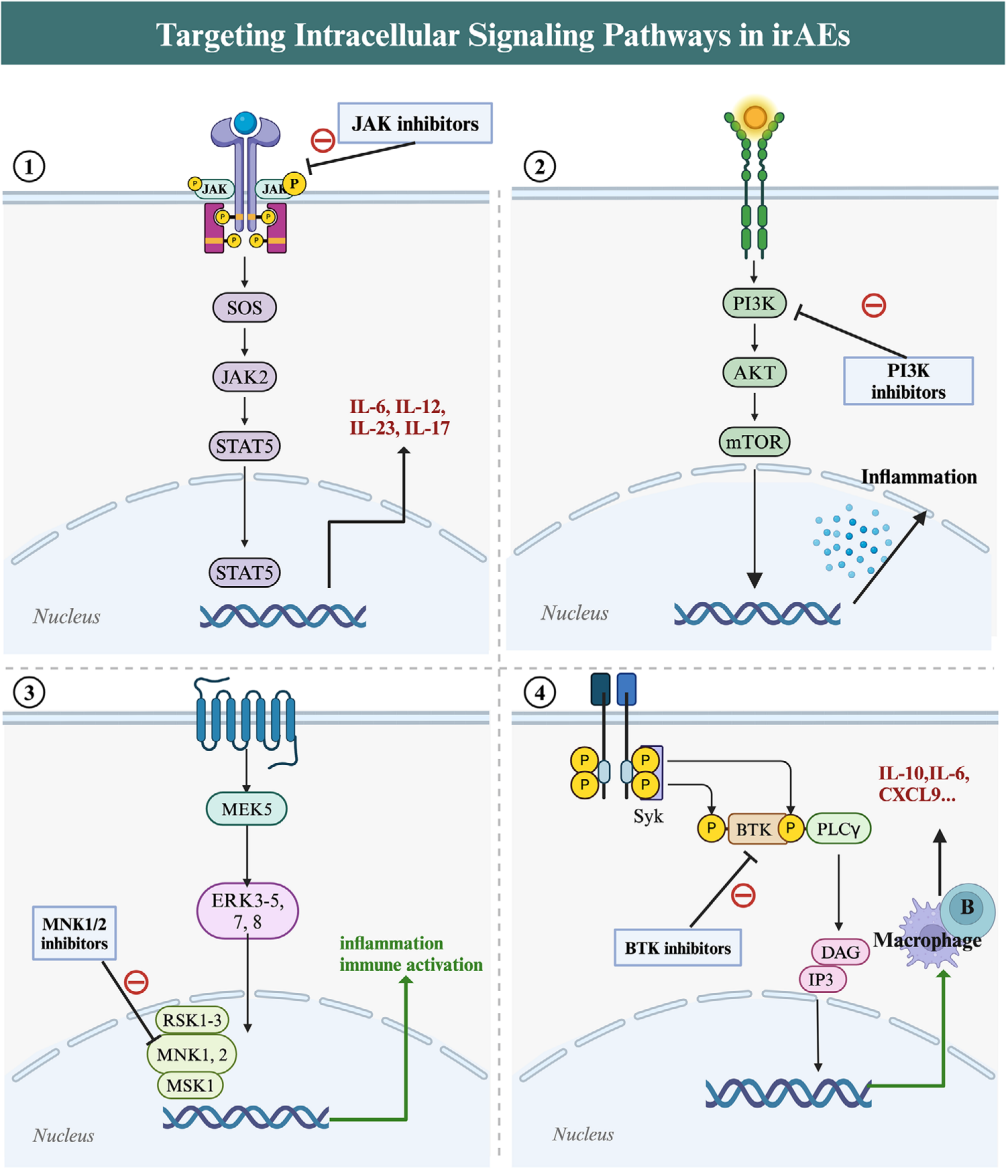
Targeting intracellular signalling pathways in immune‐related adverse events (irAEs). (1) Janus kinase–signal transducer and activator of transcription (JAK–STAT) pathway activation promotes the transcription of genes for proteins with proliferation and inflammatory functions, including those related to cytokine and chemokine production during immunotherapy (e.g., interleukin [IL]‐6, IL‐12, IL‐17 and IL‐23). Inhibitors targeting the JAK pathway can suppress these proinflammatory factors, preventing the occurrence of irAEs. (2) Phosphoinositide 3‐kinase/protein kinase B/mammalian target of rapamycin (PI3K/Akt/mTOR) pathway activation stimulates inflammation‐related mRNA translation, leading to proinflammatory cytokine production which has been associated with irAEs. PI3K inhibitors can suppress this pathway. (3) MAP kinase interacting serine/threonine kinase 1/2 (MNK1/2), which are activated by the mitogen‐activated protein kinase kinase 5 (MEK5) and extracellular signal‐regulated kinase (ERK) pathways, increase the translation of mRNAs which promote immune activation and inflammation, exacerbating irAEs. MNK1/2 inhibitors can suppress this pathway and alleviate irAEs. (4) Lck/yes novel tyrosine kinase (Lyn) and spleen tyrosine kinase (Syk) tyrosine kinases phosphorylate Bruton's tyrosine kinase (BTK), activating phospholipase C gamma (PLCγ), leading to diacylglycerol (DAG) and inositol trisphosphate (IP3) production. This stimulates macrophages and B cells to produce cytokines (IL‐6, IL‐10 and C‐X‐C motif chemokine ligand 9 [CXCL9]) related to irAEs. BTK inhibitors can mitigate overactive immune responses and reduce inflammatory infiltration.

Cytokines frequently function as cellular messengers by triggering intracellular signalling pathways, suggesting that irAE treatment might be feasible by targeting these pathways.^189^ The Janus kinase–signal transducer and activator of transcription (JAK–STAT) pathway is crucial for cellular activities and mediates the downstream effects of cytokines such as IL‐6, IL‐12, IL‐23 and IL‐17, making it a potential strategy for irAE treatment^115^, ^189^ (Figure 6). Tofacitinib, an inhibitor of this pathway, has been studied in cases of refractory colitis, myocarditis and arthritis, and showed clinical improvement in NSCLC, melanoma and other cancers.^190^, ^191^, ^192^, ^193^ Recently, Benesova et al.^194^ found that CD8+ T cells from NSCLC and melanoma patients with musculoskeletal irAEs, when treated in vitro with tofacitinib, continued to secrete cytokines and display immune‐effector cell surface markers, inhibiting lung cancer progression. These findings accentuate the potential of JAK inhibitors to augment the capacity for managing severe refractory rheumatic irAEs in NSCLC.^194^ Furthermore, the prospective evaluation of tofacitinib for addressing ICI‐related colitis in patients with NSCLC (NCT04768504) remains a topic of active investigation.^189^ However, it is imperative to acknowledge that, as of the present, large‐scale studies in this domain remain limited. Consequently, the imperative to instigate additional clinical trials, aimed at the comprehensive evaluation of these inhibitors in preventing irAEs in NSCLC is unequivocal.^189^ Beyond JAK–STAT inhibition, several other pathway inhibitors, including phosphoinositide 3‐kinase inhibitors (PI3K),^195^ MAP kinase‐interacting serine/threonine‐protein kinase 1/2 (MNK1/2)inhibitors^196^ and Bruton's tyrosine kinase (BTK) inhibitors,^197^, ^198^ have been implicated in autoimmune diseases and reducing irAEs in solid tumours ^189^ (Figure 6). Despite ongoing evaluations in preclinical models and clinical trials, these compounds hold the potential to herald a pioneering therapeutic paradigm for irAE management in NSCLC.^189^ Clearly, the advancement of therapeutic approaches focused on cell signalling pathways in NSCLC presents an exciting prospect.

## 5 CONCLUSIONS AND FUTURE PERSPECTIVES

**FIGURE 7:**
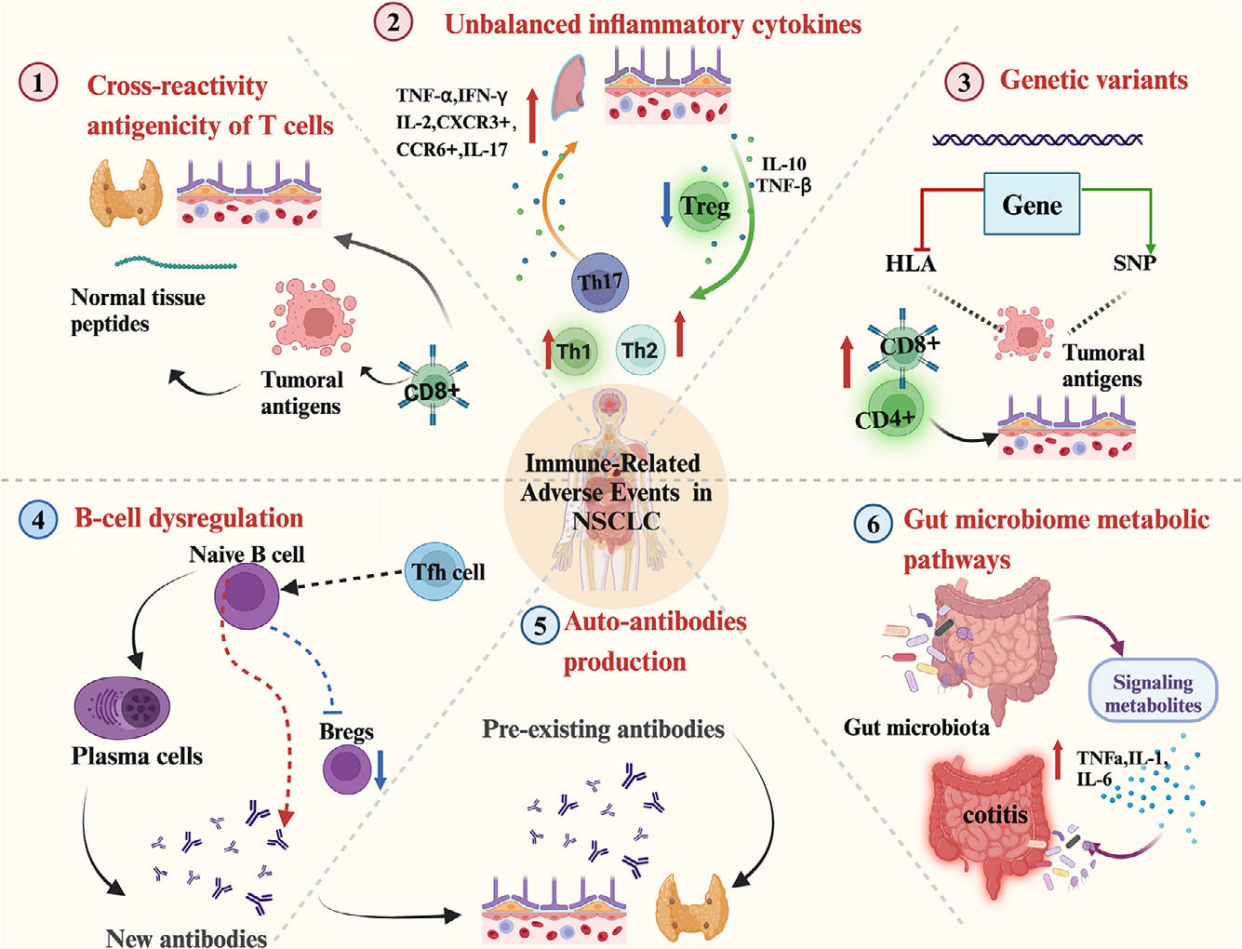
Mechanisms involved in the occurrence of immune‐related adverse events (irAEs). (1) Excessive T‐cell activation leads to cross‐reactivity between tumour and normal tissue antigens, resulting in attacks on normal tissues. (2) Increased inflammatory factor production leads to excessive local and/or systemic inflammatory responses. (3) Individual genetic polymorphisms contribute to irAE susceptibility. (4) Abnormal activation and clonal expansion of B cells can occur. (5) Production of pre‐existing or new antibodies. (6) Immune dysregulation in the gut microbiome.

ICIs have significantly transformed the landscape of cancer therapy in advanced NSCLC; however, they are still limited due to the occurrence of severe irAEs.^8^ This review has offered a detailed examination of the clinical manifestations of irAEs in NSCLC and elucidated potential therapeutic strategies rooted in mechanistic insights. Nevertheless, severe irAEs are often influenced by various factors and mediated by T cells, B cells, the host microbiome and a complex interplay of ligands, receptors and signalling pathways (Figure 7). Currently, the precise mechanisms underlying irAEs remain incompletely understood. Consequently, future research should prioritise the advancement of more efficacious therapeutic interventions in NSCLC, necessitating meticulous mechanistic preclinical and clinical investigations.

Recent strides in murine models for irAEs, exemplified by patient‐derived xenografts and humanised mouse models, have exhibited potential in devising targeted strategies aimed at ameliorating the toxicity associated with ICIs.^199^ Nonetheless, recognising the constraints of preclinical experimental models in accurately depicting irAEs is crucial.^199^ These limitations encompass inter‐species disparities in immune responses, the utilisation of allogenic cell lines instead of naturally occurring tumour growth and the inability to faithfully recapitulate genuine irAEs in murine systems.^163^, ^200^, ^201^ Concurrently, the assimilation of clinical and high‐throughput data into the formulation of precise irAEs prediction models has enriched our understanding of irAE aetiology and pathogenesis. This data‐driven approach has also empowered clinicians in discerning the selection of appropriate therapeutic regimens for clinical implementation.^163^, ^202^ Noteworthy is the pioneering work by Jing et al.,^37^ who have proffered an innovative strategy that amalgamates real‐world pharmacovigilance and molecular omics data to construct a predictive model involving lymphocyte cytosolic protein 1 (LCP1) and adenosine diphosphate‐dependent glucokinase (ADPGK) for irAEs across diverse cancer types, including NSCLC. Consequently, the accessibility and availability of human multi‐omics data have become increasingly cost‐effective, aided by open repositories, thereby facilitating widespread utilisation.^203^

Furthermore, progress in synthetic biology has opened up possibilities for utilising bioengineering and nanotechnology to develop innovative therapeutic strategies, potentially offering alternative treatments for irAEs.^204^ An example of such innovation is the design of bi/tri‐specific antibodies capable of simultaneously binding to two or three distinct entities, presenting a promising avenue for mitigating ICI‐induced toxicity while preserving efficacy.^176^ Recent advancements encompass the development of recombinant humanised PD‐L1/CTLA‐4 or PD‐1/PD‐L1 bispecific antibody Fc fusion proteins, which have evinced auspicious outcomes in preclinical investigations.^176^, ^177^ However, it behooves us to acknowledge that their therapeutic efficacy in clinical trials has yet to manifest commensurate promise in the context of NSCLC. Furthermore, alongside the exploration of novel antibodies, there is ongoing research on antibody delivery systems, specifically those with slow‐release capabilities, such as nanoparticles, virus vectors and bacteriophages.^204^ Thus, the clinical transformation of novel technology in the management of irAEs has great application prospects.

In summary, while the path forward presents numerous challenges in understanding and mitigating irAEs in NSCLC, the integration of novel therapeutic modalities promises to reshape this landscape. We anticipate that this scenario will offer significant insights for developing irAEs mitigation approaches, thereby aiding in the continuation of anti‐cancer therapies.

## AUTHOR CONTRIBUTIONS

*Design*: Xuwen Lin, Jie Yao, Xinying Xue and Mei Xie. *Writing*: Xuwen Lin, Mei Xie and Ying Jing. *Figures*: Xuwen Lin and Mei Xie. *Review and editing*: Xinying Xue, Jie Yao, Xidong Ma, Xinyu Bao, Xin Zhang, Yinguang Zhang, Yiming Liu, Wenya Han, Jialin Song and Yiran Liang. *Funding*: Xinying Xue. All the authors reviewed and approved the final manuscript.

## CONFLICT OF INTEREST STATEMENT

All authors have agreed to publish this manuscript.

## ETHICS STATEMENT

Not applicable.

## Data Availability

Not applicable.
